# Effect of chronic kidney disease on the association between hyperuricemia and new‐onset hypertension in the general Japanese population: ISSA‐CKD study

**DOI:** 10.1111/jch.14390

**Published:** 2021-11-21

**Authors:** Miki Kawazoe, Shunsuke Funakoshi, Shintaro Ishida, Chikara Yoshimura, Atsushi Satoh, Toshiki Maeda, Masayoshi Tsuji, Soichiro Yokota, Kazuhiro Tada, Koji Takahashi, Kenji Ito, Tetsuhiko Yasuno, Hideyuki Fujii, Shota Okutsu, Shigeaki Mukobara, Daiji Kawanami, Shigeki Nabeshima, Seiji Kondo, Kosuke Masutani, Hisatomi Arima

**Affiliations:** ^1^ Department of Preventive Medicine and Public Health Faculty of Medicine Fukuoka University Fukuoka Japan; ^2^ Department of Oral and Maxillofacial Surgery Faculty of Medicine Fukuoka University Fukuoka Japan; ^3^ Department of Respiratory Medicine Faculty of Medicine Fukuoka University Fukuoka Japan; ^4^ Department of Lifestyle and Welfare Information Kindai University Kyushu Junior College Fukuoka Japan; ^5^ Department of Internal Medicine Faculty of Medicine Fukuoka University Division of Nephrology and Rheumatology Fukuoka Japan; ^6^ Department of Endocrinology and Diabetes Mellitus Faculty of Medicine Fukuoka University Fukuoka Japan; ^7^ Department of General Medicine Fukuoka University Hospital Fukuoka Japan; ^8^ Nagasaki Prefecture Iki Hospital Nagasaki Japan

**Keywords:** blood pressure, chronic kidney disease, epidemiology, hypertension, hyperuricemia

## Abstract

We aimed to investigate the association between serum uric acid (SUA) level and development of hypertension as well as the interaction effect of chronic kidney disease (CKD) on this relationship in the general Japanese population. We included 7895 participants aged ≥30 years from the ISSA‐CKD study, a population‐based retrospective cohort study that used annual health check‐up data of residents from Iki Island, Japan. After the exclusion of 1881 with l < 1‐year follow‐up, 2812 with hypertension at baseline, and 165 with missing information on SUA, a total of 3037 participants were enrolled in this analysis. Participants were divided into four groups according to the quartiles of SUA level at baseline, and multivariable‐adjusted hazard ratios for new‐onset hypertension were calculated. Stratified analyses were performed for each subgroup (defined by sex, age, alcohol intake, and CKD) to assess the interaction effects. During a mean follow‐up period of 4.4 years, 943 participants developed hypertension. The first quartile group was set as the reference group, and the multivariable‐adjusted hazard ratios (95% confidence interval) for new‐onset hypertension were 1.11 (0.90–1.36) in the second quartile, 1.25 (1.02–1.54) in the third quartile, and 1.35 (1.07–1.70) in the fourth quartile compared with those in the reference group (*p* = .007 for trend). The stratified analyses showed that the association between SUA and hypertension was significantly stronger in participants with CKD than in those without CKD (*p* = .035 for interaction). SUA level is an independent risk factor for new‐onset hypertension. This tendency was significantly stronger in participants with CKD.

## 1 INTRODUCTION

Cardiovascular disease (CVD) is one of the leading causes of death in high‐income countries, including Japan, and low‐ to middle‐income countries.^1^ Hypertension is a major independent risk factor for CVD^2^; therefore, the prevention of hypertension is imperative in the field of public health to reduce the burden of CVD.

Uric acid is an end‐product of purine metabolism in humans.^3^ A number of observational studies have reported that hyperuricemia is an independent risk factor for hypertension; however, other observational studies and Mendelian randomization studies have reported no clear associations.^4^, ^5^ Previous studies have explored the association between serum uric acid (SUA) levels and the incidence of hypertension in subgroups defined by the presence of chronic kidney disease (CKD), which is highly correlated with SUA levels^6^, ^7^, ^8^ and is therefore a strong predictor of hypertension.^9^

In the present study, we aimed to determine whether hyperuricemia is an independent risk factor for the future development of hypertension in the general Japanese population. We also investigated the effects of hyperuricemia on hypertension stratified by the presence of CKD.

## 2 METHODS

### 2.1 Study design and participants

This study was a population‐based retrospective cohort study of the residents of Iki City, Nagasaki Prefecture, Japan, and used data from the Iki Epidemiological Study of Atherosclerosis and Chronic Kidney Disease (ISSA‐CKD) study, which has been described in detail previously.^10^, ^11^, ^12^, ^13^, ^14^, ^15^ Iki City is located in the north of Nagasaki Prefecture and has a population of approximately 27 000. We enrolled 7895 participants aged ≥30 years who underwent annual health check‐ups conducted by the local government of Iki City between 2008 and 2017. After the exclusion of 1881 participants with less than 1‐year follow‐up periods, 2812 participants who had been diagnosed with hypertension and/or were on blood pressure (BP)‐lowering medication at baseline, including 641 participants who fulfilled both exclusion criteria and 165 participants with missing information regarding SUA levels at baseline, were excluded. A total of 3037 participants were finally enrolled in this study. The study protocol was approved by the Fukuoka University Clinical Research and Ethics Centre (No. 2017M010).

### 2.2 Data collection

The annual health check‐ups included medical history, physical examinations, blood tests, and dipstick urine test. SUA level was determined using the enzymatic method, and hyperuricemia was defined as SUA ≥ 7.0 mg/dL (416 μmol/L).^16^

We performed two sets of analyses. In the first set, we categorized the participants into four groups according to the quartile of SUA level as follows: group 1, < 4.00 mg/dL (238 μmol/L); group 2, 4.00–4.70 mg/dL (238–279 μmol/L); group 3, 4.71–5.70 mg/dL (280–339 μmol/L); and group 4, ≥5.71 mg/dL (340 μmol/L). In the second set, we categorized the participants into two groups according to the presence of hyperuricemia (SUA < 7.0 mg/dL or ≥7.0 mg/dL [< 416 μmol/L or ≥416 μmol/L]).

We also acquired clinical information of the participants, including medical history and habitual information on current smoking, daily exercise, and alcohol intake, using a standard questionnaire. Current smoking was defined as smoking ≥100 cigarettes or regular smoking habits for more than 6 months. Daily exercise was defined as exercise for more than 30 minutes per day, two times or more per week. Daily alcohol intake was defined as regular alcohol consumption. Height and weight were measured with the participant wearing light clothes and no shoes, and body mass index (BMI, kg/m^2^) was calculated. Obesity was defined as BMI ≥25 kg/m^2^.^17^ The serum creatinine level was measured enzymatically, and the estimated glomerular filtration rate (eGFR) was calculated using the Japanese GFR equation: eGFR (mL/min/1.73 m^2^) = 194 × serum creatinine level (mg/dL) ^−1.094^ × age^−0.287^ (× 0.739 for women).^18^ CKD was defined as eGFR < 60 mL/min/1.73 m^2^ or urine protein ≥1. Plasma glucose levels were determined using an enzymatic method, and glycated hemoglobin (HbA1c) levels (NGSP: National Glycohemoglobin Standardization Program value) were determined using high‐performance liquid chromatography. Diabetes was defined as fasting glucose level of ≥7.0 mmol/L, non‐fasting glucose level of ≥11.1 mmol/L, HbA1c level of ≥6.5%,^19^ or use of glucose‐lowering therapies. Serum low‐density lipoprotein (LDL) cholesterol, high‐density lipoprotein (HDL) cholesterol, and triglyceride levels were determined enzymatically. Dyslipidemia was defined as LDL cholesterol level of ≥3.62 mmol/L, HDL cholesterol level of < 1.03 mmol/L, triglyceride level of ≥1.69 mmol/L,^20^ or current use of medications for dyslipidemia.

### 2.3 Blood pressure measurement and definition of incident hypertension

In accordance with standardized guidelines,^21^ BP was measured by trained staff on the right upper arm using a mercury, automated, or aneroid sphygmomanometer with an appropriately sized cuff after an interval of at least 5 minutes of rest with the participant seated in a chair. BP was measured twice, and the mean of the two values was used in the analysis. Elevated BP at baseline was defined as systolic BP of 130–139 mmHg and/or diastolic BP of 85–89 mmHg. Incident hypertension was defined as BP elevation to ≥140/90 mmHg^21^ or use of BP‐lowering medications, which was confirmed at the end of the follow‐up period.

### 2.4 Statistical analysis

All data were presented as the mean ± standard deviation or as n (%). Continuous variables were compared using analysis of variance, and categorical variables were compared using the chi‐squared test. Univariable and multivariable‐adjusted hazard ratios of SUA/hyperuricemia for the outcome were estimated using Cox's proportional hazards models. The effect of SUA/hyperuricemia on the outcome was compared between subgroups by adding interaction terms to the statistical models. A two‐tailed *p* value of < .05 was considered statistically significant. All statistical analyses were performed using SAS software (version 9.4).

## 3 RESULTS

Table 1 shows the baseline characteristics according to the quartile of SUA level. Participants with higher SUA levels were more likely to be male and older and had higher systolic/diastolic BP values and fasting blood glucose and higher incidences of CKD, dyslipidemia, and smoking/drinking habits. Similar patterns were observed for baseline characteristics according to the presence of hyperuricemia (Table 2).

**TABLE 1:** Baseline characteristics according to the quartiles of serum uric acid level

	Serum uric acid, mg/dL [μmol/L]				
	<4.00 [< 238]	4.00 to 4.70 [238 to 279]	4.71 to 5.70 [280 to 339]	>5.71 [≥340]	*p* for trend
No.	738	734	804	781	
Male, %	84 (11.4)	167 (22.8)	416 (51.7)	641 (84.2)	<.001
Age, y	56.3 ± 11.9	57.9 ± 11.4	58.2 ± 11.3	56.2 ± 11.6	<.001
Body mass index, kg/m^2^	21.8 ± 3.0	22.6 ± 3.2	23.3 ± 3.2	24.0 ± 3.1	<.001
Systolic blood pressure, mmHg	116.9 ± 12.5	118.8 ± 12	119.4 ± 11.8	120.5 ± 11.4	<.001
Diastolic blood pressure, mmHg	68.8 ± 8.6	70 ± 8.7	70.9 ± 8.9	72.1 ± 8.7	<.001
Serum creatinine, mg/dL	0.6 ± 0.6	0.7 ± 0.1	0.8 ± 0.5	0.9 ± 0.5	<.001
eGFR, mL/min per 1.73 m^2^	80.9 ± 15.3	77.7 ± 15	75.2 ± 14.2	73.8 ± 16	<.001
Urine protein, %	20 (2.7)	15 (2.1)	31 (3.9)	41 (5.4)	.003
CKD, %	55 (7.5)	65 (8.9)	118 (14.7)	149 (19.6)	<.001
Fasting blood glucose, mg/dL	91.4 ± 25.3	90.2 ± 16.2	94.1 ± 20.0	94.2 ± 16.1	<.001
HbA1c (NGSP), %	5.4 ± 0.6	5.4 ± 0.6	5.4 ± 0.7	5.3 ± 0.6	.043
DM, % LDL cholesterol, mg/dL	42 (5.7) 118.8 ± 31.0	38 (5.2) 124 ± 32.0	57 (7.1) 123.5 ± 31.4	44 (5.8) 125 ± 32.6	.431 < .001
HDL cholesterol, mg/dL	66.9 ± 17.1	65.8 ± 17.2	62.4 ± 16.6	57.8 ± 15.8	<.001
Triglyceride, mg/dL median (IQR)	93.3 ± 57.0 79.5 (61.0‐108.0)	97 ± 51.7 87.0 (61.0‐117.0)	112.2 ± 68.2 95.0 (70.0‐136.5)	144.3 ± 111.5 118.0 (79.0‐169.0)	.065
Hemoglobin, g/dL	13.5 ± 1.3	13.5 ± 1.3	14.1 ± 1.4	14.7 ± 1.4	<.001
Current smoker, %	82 (11.1)	100 (13.6)	183 (22.8)	257 (33.8)	<.001
Daily alcohol intake, %	58 (8.1)	73 (10.4)	167 (21.8)	279 (38.7)	<.001
Habitual exercise, %	576 (80.5)	525 (73.7)	580 (74.6)	539 (73.5)	.006

Values are expressed as mean ± standard deviation or N (%) as appropriate. eGFR: estimated glomerular filtration rate, CKD: chronic kidney disease, DM: diabetes mellitus, LDL cholesterol: low‐density lipoprotein cholesterol, HDL cholesterol: high‐density lipoprotein cholesterol. Current smoker: smoking ≥100 cigarettes or regularly smoking for more than 6 months. Daily alcohol intake: regular drinking habits. Habitual exercise: exercise for more than 30 min per day, two times or more per week.

**TABLE 2:** Baseline characteristics according to presence of hyperuricemia

	Serum uric acid, mg/dl [μmol/L]		
	<7.0 [< 416]	7.0 < = [416 < =]	*p* value
No.	2808	229	
Male, %	1092 (38.9)	216 (94.3)	<.001
Age, y	57.4 ± 11.5	54.6 ± 12.4	<.001
Body mass index, kg/m^2^	22.8 ± 3.2	24.5 ± 3.3	<.001
Systolic blood pressure, mmHg	118.78 ± 12.0	120.7 ± 11.5	.022
Diastolic blood pressure, mmHg	70.3 ± 8.7	72.8 ± 9.2	<.001
Serum creatinine, mg/dL	0.7 ± 0.5	0.9 ± 0.2	<.001
eGFR, mL/min per 1.73 m^2^	77.3 ± 15.2	71.2 ± 16.7	<.001
Urine protein, %	89 (3.2)	18 (7.9)	.002
CKD, %	331 (11.8)	56 (24.5)	<.001
Fasting blood glucose, mg/dL	92.1 ± 19.7	97.3 ± 20.8	<.001
HbA1c (NGSP), %	5.1 ± 0.7	5.2 ± 0.7	.504
DM, % LDL cholesterol, mg/dL	19 (8.3) 122.8 ± 31.8	162 (5.8) 123.1 ± 32.1	.121 .895
HDL cholesterol, mg/dL	63.7 ± 17.1	56.3 ± 14.7	<.001
Triglyceride, mg/dL median (IQR)	107.5 ± 68.3 91.0 (66.0‐129.0)	167.3 ± 146.5 127.0 (80.0‐194.0)	<.001
Hemoglobin, g/dL	13.7 ± 1.5	15.0 ± 1.4	<.001
Current smoker, %	529 (18.9)	93 (40.8)	<.001
Daily alcohol intake, %	475 (17.7)	102 (47.7)	<.001
Habitual exercise, %	2056 (75.6)	164 (75.2)	.913

Values are expressed as mean ± standard deviation or N (%) as appropriate. eGFR: estimated glomerular filtration rate, CKD: chronic kidney disease, DM: diabetes mellitus, LDL cholesterol: low‐density lipoprotein cholesterol, HDL cholesterol: high‐density lipoprotein cholesterol. Current smoker: smoking ≥100 cigarettes or regularly smoking for more than 6 months. Daily alcohol intake: regular drinking habits. Habitual exercise: exercise for more than 30 min per day, two times or more per week.

During a mean follow‐up period of 4.4 years, 943 new cases of hypertension were detected (incidence: 70 per 1000 person‐years). Table 3 shows the incidence rates and hazard ratios for the development of hypertension according to the quartiles of SUA levels. The incidence rates of hypertension (per 1000 person‐years) increased with elevation of SUA levels: 52.4 for SUA level of < 4.00 mg/dL (< 238 μmol/L), 64.7 for SUA level of 4.00–4.70 mg/dL (238–279 μmol/L), 76.0 for SUA level of 4.71–5.70 mg/dL (280–339 μmol/L), and 88.0 for SUA level of ≥5.71 mg/dL (≥340 μmol/L) (*p* < .001 for trend). This association remained significant even after adjustment for other risk factors, including age, sex, elevated BP, obesity, diabetes mellitus, dyslipidemia, current smoking, current alcohol intake, habitual exercise, and CKD: the multivariable‐adjusted hazard ratio (95% confidence interval [CI]) was 1.11 (0.90–1.36) for SUA level of 4.00–4.70 mg/dL (238–279 μmol/L), 1.25 (1.02–1.54) for SUA level of 4.71–5.70 mg/dL (280–339 μmol/L), and 1.35 (1.07–1.70) for SUA level of ≥5.71 mg/dL (≥340 μmol/L) compared with that in the reference group (< 4.00 mg/dL; < 238 μmol/L) (*p* = .007 for trend).

**TABLE 3:** Incident rates and hazard ratios for development of hypertension according to the quartiles of serum uric acid level

Serum uric acid, mg/dL [μmol/L]	<4.00 [< 238]	4.00 to 4.70 [238 to 279]	4.71 to 5.70 [280 to 339]	>5.71 [≥340]	*p* for trend
No.	738	734	804	761	
No. of events/person‐years	181/3452	216/3336	270/3553	276/3137	
Crude incidence rate (per 1,000 person‐years)	52.4	64.7	76.0	88.0	
Crude hazard ratio (95% CI)	1 (reference)	1.23 (1.01 to 1.50)	1.46 (1.21 to 1.76)	1.68 (1.39 to 2.02)	<.001
Multivariable‐adjusted hazard ratio (95% CI)*	1 (reference)	1.11 (0.90 to 1.36)	1.25 (1.02 to 1.54)	1.35 (1.07 to 1.70)	.007

* Adjusted for age, sex, elevated body pressure, obesity, diabetes mellitus, dyslipidemia, current smoking, daily alcohol intake, habitual exercise, and chronic kidney disease. 95% CI: 95% confidence interval.

Table 4 shows the incidence rates and hazard ratios for the development of hypertension according to the presence of hyperuricemia. The incidence rates of hypertension significantly increased in the participants with hyperuricemia (104.1 per 1000 person‐years) compared with that in those without hyperuricemia (67.6 per 1000 person‐years) (*p* < .001). This association was significant even after adjustment for other risk factors, including age, sex, elevated BP, obesity, diabetes mellitus, dyslipidemia, current smoking, current alcohol intake, habitual exercise, and CKD: the multivariable‐adjusted hazard ratio (95% CI) of hyperuricemia was 1.30 (1.02–1.66) (*p* = .034).

**TABLE 4:** Incident rates and hazard ratios for the development of hypertension according to the presence of hyperuricemia

Serum uric acid, mg/dL [μmol/L]	<7.0 [< 416]	7.0 < = [416 < = ]	*p* value
No.	2808	229	
No. of events/person‐years	851/12594	92/884	
Crude incidence rate (per 1,000 person‐years)	67.6	104.1	
Crude hazard ratio (95% CI)	1 (reference)	1.54 (1.24 to 1.90)	<.001
Multivariable‐adjusted hazard ratio (95% CI)*	1 (reference)	1.30 (1.02 to 1.66)	.034

* Adjusted for age, sex, elevated body pressure, obesity, diabetes mellitus, dyslipidemia, current smoking, daily alcohol intake, habitual exercise, and chronic kidney disease. 95% CI, 95% confidence interval.

Finally, we performed a stratified analysis to assess the hazard ratios for the development of hypertension in each subgroup defined by sex, age, alcohol intake, and CKD (Table 5). The effects of hyperuricemia on the development of hypertension appeared to be stronger in participants with CKD (HR: 1.67, 95% CI: 1.07–2.62) than in those without CKD (HR: 1.13, 95% CI: 0.84 –1.53) (*p* = .035 for interaction). There were no clear differences in the effects of hyperuricemia on the development of hypertension between the subgroups defined by sex, age, and alcohol intake (all *p* > .2 for interaction).

**TABLE 5:** Hazard ratios of hyperuricemia for the development of hypertension among subgroups defined by sex, age, daily alcohol intake, and CKD

		Hazard ratio for development of hypertension (95% Confidence Interval)	*p* value for interaction
Sex	Male	1.34 (1.04 to 1.74)	.209
	Female	0.59 (0.18 to 1.94)	
Age	< 65	1.05 (0.77 to 1.42)	.714
	> = 65	1.43 (0.95 to 2.16)	
Daily alcohol intake	Yes	1.40 (0.99 to 1.99)	.544
	No	1.20 (0.84 to 1.69)	
CKD	Yes	1.67 (1.07 to 2.62)	.035
	No	1.13 (0.84 to 1.53)	

CKD, chronic kidney disease.

## 4 DISCUSSION

In this large‐scale retrospective cohort study of the general Japanese population, hyperuricemia was found to be an independent risk factor for the future development of hypertension. This association was significant even after adjustment for age, sex, elevated BP, obesity, diabetes mellitus, dyslipidemia, current smoking, daily alcohol intake, habitual exercise, and CKD (multivariable‐adjusted hazard ratio: 1.30). Additionally, stratified analysis revealed that the effects of hyperuricemia on the new development of hypertension were stronger in participants with CKD than in those without CKD.

Several observational studies conducted in various populations have reported that hyperuricemia is a significant risk factor for the development of hypertension.^22^, ^23^, ^24^, ^25^ However, a limited number of longitudinal studies have shown the effects of hyperuricemia on the development of hypertension independent of the presence of CKD, which is an important confounder. The Framingham study reported significant effects of hyperuricemia on the incidence of hypertension, independent of eGFR and proteinuria: odds ratio (95% CI) of per 1 SD increase in SUA for developing hypertension was 1.17 (1.02‐1.33) in the multivariable model including eGFR and proteinuria as covariates.^26^

Our findings were in accordance with those of the Framingham study, on the other hand, this previous study did not reveal interaction between renal dysfunction and hyperuricemia for the development of hypertension. With regard to the Japanese population, a retrospective cohort study that used the data of 3,584 prehypertensive Japanese adults who underwent health check‐ups in 2004 and were reexamined in 2009 demonstrated 1.35‐fold higher risks of incident hypertension associated with hyperuricemia (defined as SUA > 7.0 mg/dL in men and ≥6.0 mg/dL in women), even after adjustment for age, sex, and BMI.^27^ The present longitudinal study of the general Japanese population confirmed the findings of the previous studies and clearly demonstrated significant linear associations between SUA and the incidence of hypertension, independent of other risk factors, including CKD.

We also demonstrated that increasing blood pressure was significantly associated with hyperuricemia even after adjusted for daily alcohol intake and the interaction for hypertension between hyperuricemia and alcohol were not significant. These results were almost accordance with recent previous study that was conducted in Japanese community‐based population (*p* for interaction between hyperuricemia and alcohol drinking: .668 in male and .811 in female).^28^ These findings suggest that the association between hyperuricemia and development of hypertension is dependent of alcohol drinking status.

In recent years, accumulating experimental studies have reported that hyperuricemia leads to vascular endothelial dysfunction, which causes BP elevation in several nonhuman animal models.^29^ It is assumed that the mechanisms underlying vascular endothelial dysfunction involve (1) sodium urate crystals, which are deposited when there is a rapid increase in SUA level, (2) oxidative stress due to nitric oxide promotion through the process of metabolism of hypoxanthine to uric acid, and (3) increased urate reabsorption via urate transporter 1, resulting in the activation of the renin–angiotensin system and vascular proliferation.^29^, ^30^, ^31^

The mechanism of interaction between hyperuricemia and CKD is not fully understood; however, Uedono and associates reported that hyperuricemia may lead to increased resistance of the renal artery and decreased glomerular filtration among participants with normal BP and renal function.^32^ Therefore, hyperuricemia that accompanies CKD could result in more severe damage to an afferent arteriole and the development of hypertension.

To the best of our knowledge, this study is the first to clarify the role of CKD in the association between hyperuricemia and the incidence of hypertension in large community‐based participants. We acknowledge there are some limitations in this study. First, the findings may have been affected by selection bias because the participants who attended the annual health check‐ups were likely to be aware of healthy behaviors. Second, we were unable to obtain information regarding the use of antihyperuricemic agents and dietary habits, which can influence the level of SUA.

## 5 CONCLUSION

Hyperuricemia is an independent risk factor for the new development of hypertension. The effects of hyperuricemia on the incidence of hypertension were larger in participants with CKD than in those without CKD, and this interaction will be needed to be verified in future research.

## CONFLICTS OF INTEREST

The authors declare no conflicts of interest associated with this manuscript.

## AUTHOR CONTRIBUTION

Conception and design of study: Shigeaki Mukobara, Daiji Kawanami, Shigeki Nabeshima, Seiji Kondo, Kosuke Masutani, and Hisatomi Arima.

Acquisition of data: Soichiro Yokota, Koji Takahashi, Kenji Ito, Tetsuhiko Yasuno, Hideyuki Fujii, and Shigeaki Mukobara.

Analysis and/or interpretation of data: Miki Kawazoe, Shunsuke Funakoshi, Shintaro Ishida, Chikara Yoshimura, Atsushi Satoh, Toshiki Maeda, Masayoshi Tsuji, Kazuhiro Tada, and Shota Okutsu.

Drafting the manuscript: Miki Kawazoe, Shunsuke Funakoshi, and Hisatomi Arima.

Revising the manuscript critically for important intellectual content: Kenji Ito, Tetsuhiko Yasuno, Daiji Kawanami, Shigeki Nabeshima, Seiji Kondo, and Kosuke Masutani.

## References

[1] Global burden of 369 diseases and injuries in 204 countries and territories, 1990–2019: a systematic analysis for the Global Burden of Disease Study 2019. The Lancet. 2020;396:(10258):1204–1222. 10.1016/S0140-6736(20)30925-9 PMC7567026 33069326

[2] O'Donnell MJ , Chin SL , Rangarajan S , et al. Global and regional effects of potentially modifiable risk factors associated with acute stroke in 32 countries (INTERSTROKE): a case‐control study. Lancet. 2016;388:761–775. 27431356 10.1016/S0140-6736(16)30506-2

[3] Johnson RJ , Bakris GL , Borghi C , et al. Hyperuricemia, acute and chronic kidney disease, hypertension, and cardiovascular disease: report of a Scientific Workshop Organized by the National Kidney Foundation. Am J Kidney Dis. 2018;71:851–865. 29496260 10.1053/j.ajkd.2017.12.009 PMC7286363

[4] Wang J , Qin T , Chen J , et al. Hyperuricemia and risk of incident hypertension: a systematic review and meta‐analysis of observational studies. PLoS One. 2014;9:e114259. 25437867 10.1371/journal.pone.0114259 PMC4250178

[5] Li X , Meng X , Timofeeva M , et al. Serum uric acid levels and multiple health outcomes: umbrella review of evidence from observational studies, randomised controlled trials, and Mendelian randomisation studies. BMJ. 2017;357:j2376. 28592419 10.1136/bmj.j2376 PMC5461476

[6] Kawashima M , Wada K , Ohta H , Terawaki H , Aizawa Y . Association between asymptomatic hyperuricemia and new‐onset chronic kidney disease in Japanese male workers: a long‐term retrospective cohort study. BMC Nephrol. 2011;12:31. 21722384 10.1186/1471-2369-12-31 PMC3146922

[7] Mok Y , Lee SJ , Kim MS , Cui W , Moon YM , Jee SH . Serum uric acid and chronic kidney disease: the Severance cohort study. Nephrol Dial Transplant. 2012;27:1831–1835. 21940488 10.1093/ndt/gfr530

[8] Obermayr RP , Temml C , Gutjahr G , Knechtelsdorfer M , Oberbauer R , Klauser‐Braun R . Elevated uric acid increases the risk for kidney disease. J Am Soc Nephrol. 2008;19:2407–2413. 18799720 10.1681/ASN.2008010080 PMC2588108

[9] Ku E , Lee BJ , Wei J , Weir MR . Hypertension in CKD: core Curriculum 2019. Am J Kidney Dis. 2019;74:120–131. 30898362 10.1053/j.ajkd.2018.12.044

[10] Maeda T , Yoshimura C , Takahashi K , et al. Usefulness of the blood pressure classification in the new 2017 ACC/AHA hypertension guidelines for the prediction of new‐onset chronic kidney disease. J Hum Hypertens. 2019;33:873–878. 31113986 10.1038/s41371-019-0198-7

[11] Yasuno T , Maeda T , Tada K , et al. Effects of HbA1c on the development and progression of chronic kidney disease in elderly and middle‐aged Japanese: iki Epidemiological Study of Atherosclerosis and Chronic Kidney Disease (ISSA‐CKD). Intern Med. 2020;59:175–180. 31554753 10.2169/internalmedicine.3242-19 PMC7008033

[12] Ito K , Maeda T , Tada K , et al. The role of cigarette smoking on new‐onset of chronic kidney disease in a Japanese population without prior chronic kidney disease: iki epidemiological study of atherosclerosis and chronic kidney disease (ISSA‐CKD). Clin Exp Nephrol. 2020;24:919–926. 32577942 10.1007/s10157-020-01914-8

[13] Miyabayashi I , Mori S , Satoh A , et al. Uric acid and prevalence of hypertension in a general population of Japanese: iSSA‐CKD Study. J Clin Med Res. 2020;12:431–435. 32655737 10.14740/jocmr4171 PMC7331868

[14] Ishida S , Kondo S , Funakoshi S , et al. White blood cell count and incidence of hypertension in the general Japanese population: iSSA‐CKD study. PLoS One. 2021;16:e0246304. 33529192 10.1371/journal.pone.0246304 PMC7853436

[15] Tada K , Maeda T , Takahashi K , et al. Association between serum uric acid and new onset and progression of chronic kidney disease in a Japanese general population: iki epidemiological study of atherosclerosis and chronic kidney disease. Clin Exp Nephrol. 2021;25(7): 751–759. 33689045 10.1007/s10157-021-02042-7

[16] Hisatome I , Ichida K , Mineo I , et al. Japanese Society of Gout and Uric & Nucleic Acids 2019 Guidelines for Management of Hyperuricemia and Gout 3rd edition. Gout Uric Nucleic Acids. 2020;44:sp‐1‐sp‐40.

[17] Examination Committee of Criteria for ‘Obesity Disease’ in J, Japan Society for the Study of O . New criteria for ‘obesity disease’ in Japan. Circ J. 2002;66:987‐992. 12419927 10.1253/circj.66.987

[18] Matsuo S , Imai E , Horio M , et al. Revised equations for estimated GFR from serum creatinine in Japan. Am J Kidney Dis. 2009;53:982–992. 19339088 10.1053/j.ajkd.2008.12.034

[19] Araki E , Goto A , Kondo T , et al. Japanese Clinical Practice Guideline for Diabetes 2019. Diabetol Int. 2020;11:165–223. 32802702 10.1007/s13340-020-00439-5 PMC7387396

[20] Kinoshita M , Yokote K , Arai H , et al. Japan Atherosclerosis Society (JAS) Guidelines for Prevention of Atherosclerotic Cardiovascular Diseases 2017. J Atheroscler Thromb. 2018;25:846–984. 30135334 10.5551/jat.GL2017 PMC6143773

[21] Umemura S , Arima H , Arima S , et al. The Japanese Society of Hypertension Guidelines for the Management of Hypertension (JSH 2019). Hypertens Res. 2019;42:1235–1481. 31375757 10.1038/s41440-019-0284-9

[22] Kuwabara M , Niwa K , Nishi Y , et al. Relationship between serum uric acid levels and hypertension among Japanese individuals not treated for hyperuricemia and hypertension. Hypertens Res. 2014;37:785–789. 24671018 10.1038/hr.2014.75

[23] Kansui Y , Matsumura K , Morinaga Y , et al. Impact of serum uric acid on incident hypertension in a worksite population of Japanese men. J Hypertens. 2018;36:1499–1505. 29746372 10.1097/HJH.0000000000001743

[24] Takase H , Kimura G , Dohi Y . Uric acid levels predict future blood pressure and new onset hypertension in the general Japanese population. J Hum Hypertens. 2014;28:529–534. 24430703 10.1038/jhh.2013.143

[25] Yokoi Y , Kondo T , Okumura N , et al. Serum uric acid as a predictor of future hypertension: stratified analysis based on body mass index and age. Prev Med. 2016;90:201–206. 27404578 10.1016/j.ypmed.2016.07.007

[26] Sundstrom J , Sullivan L , D'Agostino RB , Levy D , Kannel WB , Vasan RS . Relations of serum uric acid to longitudinal blood pressure tracking and hypertension incidence. Hypertension. 2005;45:28–33. 15569852 10.1161/01.HYP.0000150784.92944.9a

[27] Kuwabara M , Hisatome I , Niwa K , et al. Uric acid is a strong risk marker for developing hypertension from prehypertension: a 5‐year Japanese Cohort Study. Hypertension. 2018;71:78–86. 29203632 10.1161/HYPERTENSIONAHA.117.10370 PMC5730471

[28] Tatsumi Y , Asayama K , Morimoto A , et al. Hyperuricemia predicts the risk for developing hypertension independent of alcohol drinking status in men and women: the Saku study. Hypertens Res. 2020;43:442–449. 31776471 10.1038/s41440-019-0361-0

[29] Mallat SG , S AlKattar , Tanios BY , Hyperuricemia JurjusA . Hypertension, and chronic kidney disease: an emerging association. Curr Hypertens Rep. 2016;18:74. 27696189 10.1007/s11906-016-0684-z

[30] Sanchez‐Lozada LG , Tapia E , Santamaria J , et al. Mild hyperuricemia induces vasoconstriction and maintains glomerular hypertension in normal and remnant kidney rats. Kidney Int. 2005;67:237–247. 15610247 10.1111/j.1523-1755.2005.00074.x

[31] Anzai N , Ichida K , Jutabha P , et al. Plasma urate level is directly regulated by a voltage‐driven urate efflux transporter URATv1 (SLC2A9) in humans. J Biol Chem. 2008;283:26834–26838. 18701466 10.1074/jbc.C800156200

[32] Uedono H , Tsuda A , Ishimura E , et al. U‐shaped relationship between serum uric acid levels and intrarenal hemodynamic parameters in healthy subjects. Am J Physiol Renal Physiol. 2017;312:F992–F997. 28249837 10.1152/ajprenal.00645.2016

